# Quality of life and physical/psychosocial factors in children and adolescents with orthostatic intolerance

**DOI:** 10.1186/s13030-023-00278-1

**Published:** 2023-06-12

**Authors:** Yoshie Shigeyasu, Ayumi Okada, Chikako Fujii, Chie Tanaka, Akiko Sugihara, Makiko Horiuchi, Takashi Yorifuji, Hirokazu Tsukahara

**Affiliations:** 1 Department of Pediatrics, Okayama University Graduate School of Medicine, Dentistry and Pharmaceutical Sciences, 2-5-1, Shikata-Cho, Kita-Ku, Okayama, 7008558 Japan; 2 Clinical Psychology section, Department of Medical Support, Okayama University Hospital, 2-5-1, Shikata-Cho, Kita-Ku, Okayama, 7008558 Japan; 3 Department of Epidemiology, Okayama University Graduate School of Medicine, Dentistry and Pharmaceutical Sciences, 2-5-1, Shikata-Cho, Kita-Ku, Okayama, 7008558 Japan

**Keywords:** Quality of life, Orthostatic intolerance, Psychosomatic factors, School nonattendance, Postural orthostatic tachycardia syndrome, School-aged children, Adolescence

## Abstract

**Background:**

Orthostatic intolerance (OI), which is common in children and adolescents, negatively impacts their quality of life (QOL) due to physical symptoms that limit work, school, and daily activities. The purpose of this study is to explore the association of physical and psychosocial factors with QOL scores in children and adolescents with OI.

**Methods:**

A cross sectional observational study was conducted. The study participants included 95 Japanese pediatric patients of age 9–15 years who were diagnosed with OI from April 2010 to March 2020. The QOL scores and the QOL T-scores of children with OI assessed using the KINDL-R questionnaire at the initial visit were compared with conventional normative data. The associations of physical and psychosocial factors with the QOL T-scores were examined using multiple linear regression.

**Results:**

Pediatric patients with OI had significantly lower QOL scores than healthy children in both elementary (50.7 ± 13.5 vs. 67.9 ± 13.4, *p* < 0.001) and junior high schools (51.8 ± 14.6 vs. 61.3 ± 12.6, *p* < 0.001). This finding was observed in the physical, mental, self-esteem, friends, and school domains. Total QOL scores were significantly associated with school nonattendance (β =  − 3.2, 95% confidence interval [CI] =  − 5.8 to − 0.5, *p* = 0.022) and poor relationship with school (β =  − 5.0, 95% CI =  − 9.8 to − 0.4, *p* = 0.035).

**Conclusions:**

These results suggest that the assessment of QOL, including both physical and psychosocial aspects, especially school factors, needs to be implemented earlier in children and adolescents with OI.

## Background

Orthostatic intolerance (OI), which is common in children and adolescents, can be defined as having difficulty tolerating the upright posture because of symptoms that abate when the patient returns to the supine position [1, 2]. The clinical symptoms are diverse, including chronic fatigue, headache, dizziness, exercise intolerance, and syncope because of dysregulation of the autonomic nervous system and cardiovascular compensatory mechanisms [2–4]. The subtypes of OI include postural orthostatic tachycardia syndrome (POTS), orthostatic hypotension (OH), and vasovagal syncope (VVS), which are evaluated based on chronic symptoms and orthostatic tests, either the active standing test or the head-up tilt test [2, 5, 6].

OI is not life-threatening and 86% of patients with POTS reported improved OI symptoms after 5 years from diagnosis [7]. In contrast, it can severely impair work, school, and daily activities [7–9] and reduce QOL [8–13]. Early psychological support is likely to be important in preventing QOL deterioration and associated secondary factors, such as reactive depression, avoidance, and physical deconditioning [14].

In adult studies, multiple physical symptoms have been implicated in reduced QOL [10–12]. Compared to physical factors, the relationship between QOL and psychosocial factors in patients with OI has not been fully elucidated. Moreover, all previous studies included adult or adolescent patients, and the QOL in children and adolescents with OI remains unclear.

This study aimed to quantify QOL scores and determine the association of physical and psychosocial factors with QOL in children and adolescents with OI.

## Methods

### Participants and measures

In this cross sectional observational study, the medical records of 95 outpatients aged between 9–15 years who were diagnosed with OI at the Department of Child Psychosomatic Medicine in the Okayama University Hospital, Japan from April 2010 to March 2020 were reviewed. Basic clinical information at the initial visit was extracted from the electronic medical records, including age, sex, school year, the period from the onset, subtypes and severity of OI, experiences already diagnosed with OI, and comorbidities.

OI was diagnosed based on symptoms and the active standing test, defined by the Japanese Clinical Guidelines [5, 15]. Other disorders that could cause similar clinical symptoms of orthostatic intolerance were excluded based on the guidelines. In the active standing test, blood pressure and heart rate were measured three times after 10 min of rest in the supine position and five times upon standing (after 1, 3, 5, 7, and 10 min), and the initial recovery time of blood pressure from the fall after standing up was measured. We evaluated subtypes depending on the result of the active standing test. The subtypes included the following four forms [5]: (1) instantaneous orthostatic hypotension (INOH), (2) POTS, (3) neurally mediated syncope (NMS), and (4) delayed orthostatic hypotension (delayed OH). If the patients had orthostatic symptoms but did not meet the four subtypes, cerebral blood flow was measured using near-infrared spectroscopy in the active standing test. The orthostatic cerebral hypoperfusion type was evaluated in which cerebral oxygenated hemoglobin decreases by more than 4 μmol/l [15]. According to the severity, it is divided into severe or mild forms. In the Japanese guidelines, only INOH and POTS are defined as severe and mild forms, depending on the degree of heart rate increase or initial recovery time of blood pressure using the active standing test [5]. Participants with severe forms of INOH and POTS were assigned to the severe group, whereas those with mild forms of INOH and POTS, delayed OH, and orthostatic cerebral hypoperfusion were assigned to the mild group. Physical diseases as comorbidities were diagnosed based on the ICD-10 [16]. Psychiatric disorders and neurodevelopmental disorders as comorbidities were diagnosed using the fifth edition of the Diagnosis and Statistical Manual of Mental Disorders [17].

### Quality of life questionnaire

We used the Japanese version of KINDL-R to assess the health-related QOL [18, 19]. The self-administered KINDL-R questionnaire [20] is a German-language tool designed to study the health-related QOL in children and adolescents by evaluating the following subscales: physical wellbeing, emotional wellbeing, self-esteem, and family-, friend-, and school-related QOL. Each of these six subscales consists of four items that are each rated on a 5-point Likert scale. The mean scores for each subscale and a total QOL score are calculated on a scale from 0 to 100, with higher values corresponding to better/higher QOL. The KINDL-R questionnaire is validated in Japanese. The following versions of the KINDL-R questionnaire are available as self-report measures for different age groups: Kid-KINDL [18] for children in elementary schools, and Kiddo-KINDL [19] for adolescents in junior high schools (Japanese version). The data were standardized for 3702 and 2306 students, respectively, out of 4607 students from 19 elementary schools and 2926 students from 9 junior high schools in Japan, who responded that they had no disease currently under treatment at a hospital.

Children and adolescents filled in the KINDL-R questionnaire at the first visit. A total of 21 patients out of 95 did not answer enough items on each subscale. Thus, blank subscale and total QOL scores could not be calculated, and only subscale scores answered were included in the results. We also converted the QOL scores to age- and sex-matched T-scores with a mean of 50 and standard deviation (SD) of 10 using reference values from 6008 healthy Japanese children and adolescents [3702 elementary school students by Kid-KINDL [18] and 2306 junior high school students by Kiddo-KINDL [19].

### Evaluation of psychosocial factors

The following psychosocial factors were selected from the reference to the psychosocial evaluation for pediatric psychosomatic disorders [21]. The psychosocial status, which includes the number of parents living with children, family psychiatric disorders, academic grades, relationship with school, school nonattendance, social problems, and activities outside of school, were taken from the parents’ medical interview sheet at the time of the initial visit. The number of parents living with children was defined by the number of parents living together. Academic grades and relationship with school were divided into good or not-good based on the parents’ subjective responses in the initial medical questionnaire. Social problems were divided by whether or not they had bullying or interpersonal problems. School nonattendance was considered if the OI patient was absent from school for more than half of the past month.

### Statistical analysis

After the descriptive analysis, we used a t-test to compare the (raw) QOL scores as well as the QOL T-scores between children with OI and healthy children. The reference values of the healthy children were obtained from the above-mentioned references [18, 19] and almost normally distributed [22]. We also compared the QOL T-scores separated by demographic groups (i.e., sex, school levels, and comorbidity) among children with OI and performed the t-test to examine the difference. We then examined the associations of physical and psychosocial factors with the QOL T-scores using multiple linear regression. Multiple regression analysis was performed with QOL T-scores as dependent variables and each psychosocial factor (binary) as independent variables, controlling for sex (binary), school levels (binary: elementary school; junior high school), and presence of comorbidities (binary). The variance inflation factors (VIFs) were tested to identify the degree of multicollinearity in all models. The VIFs of the nine items as physical and psychosocial factors, as well as three items related to characteristics were less than 1.5, indicating no multicollinearity. We estimated regression coefficients (β) with 95% confidence intervals (CIs). All statistical analyses were performed using JMP 10 (SAS Institute Inc., Cary, NC, USA). P values less than 0.05 were considered statistically significant.

### Ethical approval

This study was approved by the Ethics Committee of the Okayama University Graduate School of Medicine, Dentistry, and Pharmaceutical Science and Okayama University Hospital (No. 2006–005).

## Results

### Patient demographics and orthostatic intolerance status

**Fig. 1:**
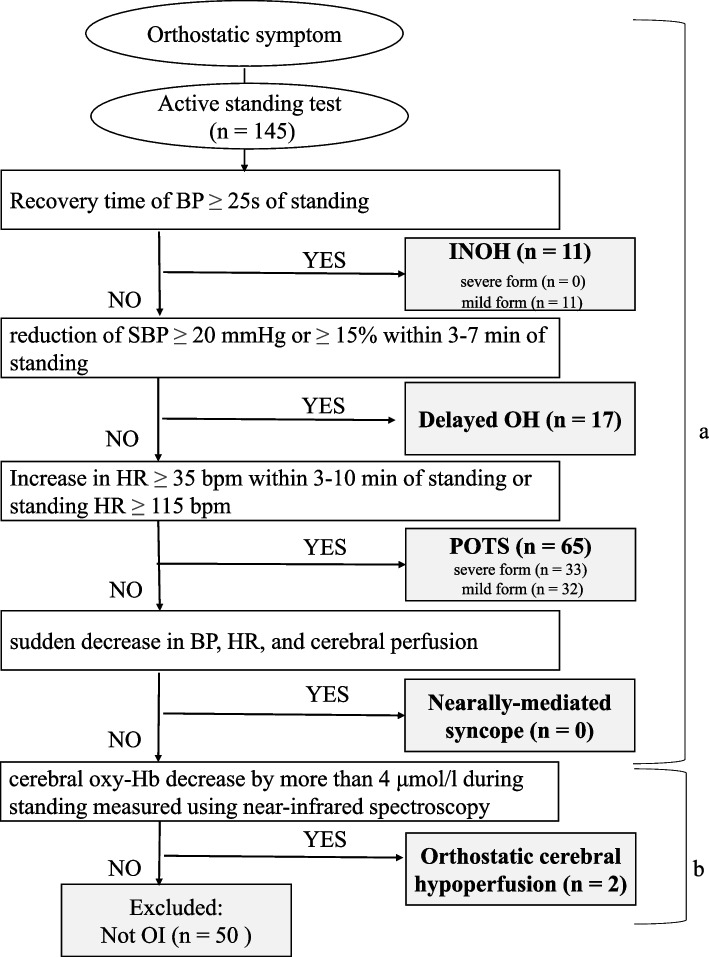
Diagnostic criteria for orthostatic intolerance in this study. a: based on reference 5. b: based on references 15. BP, blood pressure; INOH, instantaneous orthostatic hypotension; SBP, systolic blood pressure; delayed OH, delayed orthostatic hypotension; HR, heart rate; POTS, postural orthostatic tachycardia syndrome; OI, orthostatic intolerance

A total of 95 participants were included in this study (Fig. 1). Patient demographics and OI data are presented in Table 1. The average age was 12.9 ± 1.4 years, 47 were male, and 19 were enrolled in elementary school. The mean period from onset to the first visit was 9.9 ± 8.3 months. Comorbidities with physical diseases in 28 cases include irritable bowel syndrome, allergic rhinitis, migraine, atopic dermatitis, functional dyspepsia, chronic sinusitis, obesity, ventricular septal defect, scoliosis, precocious puberty, nephropathy IgA, and Marfan syndrome.

**Table 1:** Demographic and clinical characteristics of the patients with OI

Characteristics	
Patients, n	95
Age (years), Mean (SD)	12.9 (1.4)
Sex, n	
Male	47
Female	48
School level, n	
Elementary school (9–12 years)	19
Junior high school (13–15 years)	76
Period until the first visit (months), Mean (SD)	9.9 (8.3)
Experience already diagnosed with OI, n	
Yes	43
No	52
Comorbidities, n	
Physical diseases	28
Psychiatric disorders	7
Neurodevelopmental disorders	40

*OI* Orthostatic intolerance, *SD* Standard deviation

### Quality of life and T-scores

Mean and SD for each of the separate KINDL-R subscales and total QOL scores and T-scores are shown in Table 2. Patients with OI had significantly lower QOL scores than healthy children, in both elementary (50.7 ± 13.5 vs. 67.9 ± 13.4, *p* < 0.001) and junior high school students (51.8 ± 14.6 vs. 61.3 ± 12.6, *p* < 0.001). In the six subscales, the QOL T-score for physical wellbeing was the lowest, 38.2 ± 14.3. Emotional wellbeing, self-esteem, friend-, and school-related QOL T-scores were significantly lower than the healthy group.

**Table 2:** KINDL-R QOL raw scores and T-scores for OI patients and healthy controls

	n	Physical	Emotional	Self-esteem	Family	Friends	School	Total
		Mean (SD)						
QOL scores (Kid KINDL: elementary school students)								
OI	19	41.0 (18.2)^#^	67.8 (18.1)^**^	25.4 (28.4)^#^	67.4 (20.3)	45.1 (18.4)^#^	44.1 (22.2)^**^	50.7 (13.5)^#^
Healthy controls^b^	3702	77.2 (16.9)	79.3 (17.5)	53.7 (24.6)	68.9 (19.6)	69.8 (18.0)	58.4 (20.0)	67.9 (13.4)
QOL scores (Kiddo KINDL: junior high school students)								
OI	76	49.1 (25.3)^#^	67.3 (22.1)^*^	20.6 (21.5)^#^	71.5 (21.2)	60.6 (24.9)^#^	44.5 (22.8)^#^	51.8 (14.6)^#^
Healthy controls^b^	2306	65.9 (17.8)	72.3 (17.7)	35.4 (22.1)	66.7 (21.1)	71.0 (17.0)	52.6 (12.6)	61.3 (12.6)
T-scores^a^								
OI	95^c^	38.2 (14.3)^#^	44.1 (12.6)^#^	42.3 (9.7)^#^	51.3 (10.1)	42.1 (14.5)^#^	45.0 (11.7)^#^	41.2 (11.2)^#^
Healthy controls^b^	6008	50 (10)	50 (10)	50 (10)	50 (10)	50 (10)	50 (10)	50 (10)

Significant difference for the t-test: *p < 0.05, **p < 0.01, #*p* < 0.001

*QOL* Quality of life, *OI* Orthostatic intolerance, *SD* Standard deviation

^a^T-score was calculated and compared with age- and sex-matched normative data

^b^Healthy controls data for reference 18 and 19

^c^QOL scores include missing data; of 95, 1 in physical, 2 in emotional, 1 in self-esteem, 2 in family, 8 in friends, 16 in school, and 21 in total are missing

Females reported lower QOL T-scores in friend- and school-related subscales compared with males. Elementary school students reported more impairment in physical and friend-related QOL compared to junior high school students. Patients with comorbid neurodevelopmental disorders had lower emotional, self-esteem, family, and total QOL scores than those without comorbidity (Table 3).

**Table 3:** QOL T-score and demographic variable of OI patients

	n	Physical	Emotional	Self-esteem	Family	Friends			School	Total
		Mean (SD)								
Sex										
Male	47	39.2 (13.8)	42.2 (13.5)	42.4 (10.0)	50.7 (10.9)		45.6 (12.9)	49.1 (10.9)		42.5 (10.7)
Female	48	37.2 (15.1)	46.1 (11.6)	42.1 (9.6)	52.4 (9.4)		38.3 (15.5)^*^	39.5 (10.7)^**^		39.3 (12.1)
School level										
Elementary school	19	29.0 (10.8)^**^	42.5 (11.0)	40.2 (11.0)	48.6 (10.5)		36.6 (10.4)^**^	44.3 (10.6)		37.8 (9.9)
Junior high school	76	40.6 (14.3)	44.6 (13.1)	42.8 (9.5)	52.3 (10.0)		43.6 (15.2)	45.1 (12.1)		42.1 (11.6)
Comorbidity with physical diseases										
Yes	28	39.6 (13.4)	41.7 (13.1)	42.7 (9.2)	48.7 (10.3)^*^		42.1 (13.7)	46.1 (11.8)		40.3 (10.9)
No	67	37.6 (14.9)	45.1 (12.5)	42.1 (10.1)	52.8 (9.9)		42.0 (15.1)	44.5 (11.8)		41.5 (11.6)
Comorbidity with psychiatric disorders										
Yes	7	44.0 (6.8)	44.6 (10.0)	43.7 (7.3)	54.5 (9.7)		23.7 (15.7)^*^	48.5 (3.7)		39.3 (12.2)
No	88	37.8 (14.8)	44.1 (12.9)	42.2 (10.0)	51.3 (10.2)		43.2 (13.9)	44.8 (12.0)		41.3 (11.4)
Comorbidity with neurodevelopmental disorders										
Yes	40	37.6 (14.7)	41.3 (12.6)^*^	39.8 (9.5)^*^	48.3 (10.5)^*^		41.4 (13.0)	42.8 (10.0)		38.3 (11.0)^*^
No	55	38.6 (14.3)	46.2 (12.4)	44.1 (9.7)	53.9 (9.3)		42.5 (15.9)	46.7 (12.8)		43.4 (11.2)

Significant difference for t-test, * *p* < 0.05, ** *p* < 0.01

*QOL* Quality of life, *OI* Orthostatic intolerance, *SD* Standard deviation

### Physical and psychosocial factors associated with quality of life

Table 4 showed the associations of physical and psychosocial factors with the QOL T-scores. Physical QOL was affected by experiences already diagnosed with OI (β =  − 2.9, 95%CI =  − 5.8 to − 0.1, *p* = 0.044) and school nonattendance (β =  − 3.8, 95%CI =  − 6.9 to − 0.7, p = 0.016). Emotional QOL was associated with school nonattendance (β =  − 2.9, 95%CI =  − 5.7 to − 0.1, *p* = 0.048) and social problem (β =  − 3.2, 95%CI =  − 6.3 to − 0.2, *p* = 0.039). Friend-related and total QOL was affected by the relationship with school (friend: β =  − 10.7, 95%CI =  − 15.3 to − 6.1, *p* < 0.001; total QOL: β =  − 5.0, 95%CI =  − 9.8 to − 0.4, *p* = 0.035) and school nonattendance (friend: β =  − 3.7, 95%CI =  − 7.0 to − 0.4, *p* = 0.029; total QOL: β =  − 3.2, 95%CI =  − 5.8 to − 0.5, *p* = 0.022).

**Table 4:** Multiple linear regression analysis for QOL T-scores based on physical and psychosocial factors

			Physical		Emotional		Self-esteem		Family		Friends		School		Total	
		n	T-score	β (95%CI)^a^	T-score	β (95%CI)^a^	T-score	β (95%CI)^a^	T-score	β (95%CI)^a^	T-score	β (95%CI)^a^	T-score	β (95%CI)^a^	T-score	β (95%CI)^a^
**Disease factor**																
Severity of OI	Mild	62	39.2		45.0		42.9		51.3		44.1		44.6		41.5	
	Severe	33	36.5	-0.7(-3.8, 2.4)	42.7	-0.7(-3.4, 2.0)	41.1	-0.8(-3.0, 1.4)	51.8	0.7(-1.5, 2.9)	38.6	-3.0(-6.1, 0.2)	45.6	-0.6(-3.2, 2.0)	40.5	-0.8(-3.5, 2.0)
Experiences already diagnosed with OI	No	52	41.0		41.9		41.8		49.3		41.7		44.5		39.8	
	Yes	43	35.0	-2.9(-5.8, -0.1)*	46.9	2.0(-0.6, 4.6)	42.9	0.4(-1.7, 2.5)	54.1	1.9(-0.2, 4.0)	42.5	0.2(-3.0, 3.3)	45.6	0.1(-2.5, 2.6)	43.0	1.1(-1.6, 3.8)
**Family factor**																
Number of parents living with children	two parents	74	39.5		44.2		42.5		52.3		42.3		46.2		41.8	
	a single parent	21	33.8	-2.8(-6.2, 0.7)	43.9	0.5(-2.7, 3.7)	41.6	-0.2(-2.7, 2.3)	48.5	-1.4(-3.9, 1.1)	41.3	-0.2(-3.9, 3.6)	41.1	-2.4(-5.2, 0.5)	38.9	-1.2(-4.3, 1.9)
Family with psychiatric disorders	No	78	37.5		45.0		42.4		52.5		41.1		44.7		41.7	
	Yes	17	41.4	1.3(-2.4, 5.0)	40.4	2.0(-5.5, 1.2)	41.6	-0.4(-3.1, 2.2)	46.9	-2.8(-5.4, -0.2)*	41.8	-0.3(-4.3, 3.7)	46.5	1.0(-2.3, 4.3)	38.7	-1.7(-5.2, 1.7)
**School factor**																
Academic grade	Good	72	37.9		44.3		43.0		52.4		42.8		46.2		42.0	
	Not good	23	39.2	-0.4(-3.9, 3.2)	43.5	-0.1(-3.3, 3.1)	40.0	-1.6(-4.0, 0.8)	48.6	-1.8(-4.3, 0.7)	39.9	-1.6(-5.3, 2.1)	41.1	-1.3(-4.3, 1.7)	38.0	-1.5(-4.8, 1.9)
Relationship with school	Good	86	38.6		44.5		42.6		51.6		44.1		45.1		41.9	
	Not good	9	34.2	-3.6(-8.5, 1.3)	40.6	-3.0(-7.4, 1.4)	39.1	-2.2(-5.7, 1.3)	50.5	-1.6(-5.1, 1.9)	24.7	-10.7(-15.3, -6.1)**	43.3	-0.3(-5.0, 4.3)	33.0	-5.0(-9.8, -0.4)*
School nonattendance	No	31	42.3		46.2		43.5		51.5		47.2		46.3		44.6	
	Yes	64	36.2	-3.8(-6.9, -0.7)*	43.1	-2.9(-5.7, -0.1)*	41.7	-1.2(-3.4, 1.1)	51.6	-0.9(-3.2, 1.4)	39.5	-3.7(-7.0, -0.4)*	44.2	-0.2(-2.8, 2.4)	39.0	-3.2(-5.8, -0.5)*
**Social factor**																
Social problem	No	73	39.2		45.4		42.7		52.1		43.7		46.0		41.9	
	Yes	22	35.2	-2.4(-5.8, 1.1)	40.1	-3.2(-6.3, -0.2)*	40.8	-0.9(-3.4, 1.5)	49.6	-1.5(-4.0, 1.0)	36.3	-2.9(-6.7, 0.8)	41.4	-0.9(-3.9, 2.0)	38.4	-1.4(-4.6, 1.9)
Activities outside of school	No	44	38.0		43.7		42.1		50.0		41.0		43.8		40.4	
	Yes	51	38.4	0.7(-2.2, 3.6)	44.6	0.1(-2.5, 2.8)	42.4	0.1(-1.9, 2.2)	52.9	1.3(-0.8, 3.3)	43.0	1.1(-2.0, 4.2)	46.0	0.5(-2.0, 3.0)	41.8	0.5(-2.2, 3.1)

Significant difference, *p<0.05, **p<0.01

Missing value are included : of 95 patients, 1 in physical, 2 in emotional, 1 in self-esteem, 2 in family, 8 in friends, 16 in school, and 21 in total are missing.

*QOL* Quality of life, *OI* Orthostatic intolerance

^a^Adjusted for sex, school levels (elementary school or junior high school) and presence of comorbidities

## Discussion

### Quality of life in children and adolescents with orthostatic intolerance

To our knowledge, this study is the first report that quantifies the QOL scores in children and adolescents with OI. Similar to the results of several previous studies in adults [8, 9, 12, 13], the QOL of the pediatric patients with OI was significantly lower than that of the healthy group. Compared with other chronic diseases [headache [23], type 1 diabetes [24], asthma [25]] assessed by QOL with KINDL-R, low scores in OI are noted.

Several previous studies have examined the QOL of physical versus mental parameters in adult patients with POTS [8–14]. Physical QOL in patients with POTS is reduced in these studies. One of them reported that physical QOL scores are as low as that in chronic obstructive pulmonary disease and congestive heart failure [9]. On the contrary, mental QOL remains controversial. Some studies revealed that mental parameters did not significantly differ between patients and healthy subjects [9, 13], and other studies showed significant impairment in both physical and mental QOL in patients compared with healthy subjects [8, 10–12].

In this study, we investigated six parameters of QOL. QOL in children with OI was significantly reduced in multiple parameters, including physical wellbeing, emotional wellbeing, self-esteem, friend, and school parameters. The results showed that OI had a multidisciplinary impact on child and adolescent patients. Moreover, elementary school students and the female group represented lower QOL scores. Younger adult patients with POTS were reported to have lower functional capacity than expected compared to older patients [13]. Similarly, younger children can be more susceptible to environmental influences.

Therefore, we suggest that QOL should be assessed as the first step to evaluating both the physical and the psychosocial aspects.

### Association of physical and psychosocial factors with quality of life

In recent years, multiple physical factors associated with QOL in adult patients with POTS were reported. For instance, QOL scores were linked to some symptoms, such as sleep impairment, lightheadedness, dizziness, headache, and concentration difficulties [10–12]. Additionally, even patients with mild OI symptoms had significantly reduced QOL [10]. No correlation of QOL scores with heart rate or blood pressure was reported [10, 11]. In this study, there was no clear association between QOL and severity of OI including the degree of heart rate increase or initial recovery time of blood pressure.

In the adult field, there have been few studies on psychosocial factors related to QOL in patients with OI. In this study, five factors were found to be significantly related to QOL: experiences already diagnosed with OI, family with psychiatric disorders, relationship with school, school nonattendance, and social problems. In particular, school-related factors severely impaired the QOL of multiple parameters and total scores. One reason for this is that pediatric patients with OI who have difficulty attending school are deprived of opportunities to learn and built friendships. As a result, their academic performance and social functioning may be adversely affected. This is thought to be a characteristic of pediatric patients with OI. It has been reported that children with psychosomatic disorders have lower self-esteem [26], especially in the academic area [27]. Therefore, in addition to physical treatment, a psychosocial approach should be emphasized to improve the QOL of OI patients.

In this study, 42.1% of OI patients had comorbid neurodevelopmental disorders. Neurodevelopmental disorders such as autism spectrum disorder (ASD) and attention-deficit/hyperactivity disorder (ADHD) have been reported to be associated with a high prevalence of comorbidity with migraine, asthma, and other physical disorders [28, 29]. In OI, adult patients with POTS were reported to score significantly higher than controls on the inattention and ADHD subscales [30]. ASD is linked to autonomic dysfunction [31]. However, the relationship between OI and neurodevelopmental disorders has not been well reported. The poor QOL in multiple domains could be the result of the addition of physical symptoms and difficulties caused by OI to the difficulties in their lives caused by neurodevelopmental disorders. Further investigation into the association of OI with neurodevelopmental disorders is therefore needed.

### Limitations of this study and future directions

There are several limitations to this study. First, all patients were recruited from a single hospital, so there may be a problem with the generalizability of the findings. Since this hospital deals with referral patients, the target patients may be severely ill and have significant psychosocial factors. Second, about one-fourth of the participants did not completely answer the KINDL-R questionnaire, resulting in missing values. This may have resulted in overestimation or underestimation of QOL scores. Third, in this study, we did not consider the parental economic status or educational level as psychosocial factors. Further research is needed in the future.

## Conclusions

This study found that QOL in children and adolescents with OI was significantly lower scores than in healthy children in multiple categories, including physical, emotional, self-esteem, friend, and school. Moreover, investigations of psychosocial factors revealed that not-good relationships with school, nonattendance school, social problems, and family psychiatric disorders were associated with reduced QOL. These findings suggest that the assessment of QOL including physical and psychosocial factors is valuable. In pediatrics OI, particular attention should be paid to school-related factors.

## Data Availability

Not applicable.

## Abbreviations

ADHD: Attention-deficit/hyperactivity disorder
ASD: Autism spectrum disorder
CI: Confidence interval
delayed OH: Delayed orthostatic hypotension
INOH: Instantaneous orthostatic hypotension
NMS: Neurally mediated syncope
OH: Orthostatic hypotension
OI: Orthostatic intolerance
POTS: Postural orthostatic tachycardia syndrome
QOL: Quality of life
SD: Standard deviation
VVS: Vasovagal syncope
VIF: Variance inflation factor
